# Mitochondrial “power” drives tamoxifen resistance: NQO1 and GCLC are new therapeutic targets in breast cancer

**DOI:** 10.18632/oncotarget.15852

**Published:** 2017-03-02

**Authors:** Marco Fiorillo, Federica Sotgia, Diego Sisci, Anna Rita Cappello, Michael P. Lisanti

**Affiliations:** ^1^ The Department of Pharmacy, Health and Nutritional Sciences, The University of Calabria, Cosenza, 87100, Italy; ^2^ The Paterson Institute, University of Manchester, Withington, M20 4BX, United Kingdom; ^3^ Translational Medicine, School of Environment and Life Sciences, Biomedical Research Centre, University of Salford, Greater Manchester, M5 4WT, United Kingdom

**Keywords:** tamoxifen resistance, endocrine therapy, mitochondria, drug resistance, breast cancer

## Abstract

Here, we identified two new molecular targets, which are functionally sufficient to metabolically confer the tamoxifen-resistance phenotype in human breast cancer cells. Briefly, ~20 proteins were first selected as potential candidates, based on unbiased proteomics analysis, using tamoxifen-resistant cell lines. Then, the cDNAs of the most promising candidates were systematically transduced into MCF-7 cells. Remarkably, NQO1 and GCLC were both functionally sufficient to autonomously confer a tamoxifen-resistant metabolic phenotype, characterized by i) increased mitochondrial biogenesis, ii) increased ATP production and iii) reduced glutathione levels. Thus, we speculate that pharmacological inhibition of NQO1 and GCLC may be new therapeutic strategies for overcoming tamoxifen-resistance in breast cancer patients. In direct support of this notion, we demonstrate that treatment with a known NQO1 inhibitor (dicoumarol) is indeed sufficient to revert the tamoxifen-resistance phenotype. As such, these findings could have important translational significance for the prevention of tumor recurrence in ER(+) breast cancers, which is due to an endocrine resistance phenotype. Importantly, we also show here that NQO1 has significant prognostic value as a biomarker for the prediction of tumor recurrence. More specifically, higher levels of NQO1 mRNA strongly predict patient relapse in high-risk ER(+) breast cancer patients receiving endocrine therapy (mostly tamoxifen; H.R. > 2.15; *p* = 0.007).

## INTRODUCTION

Drug-resistance, and the resulting treatment failure, are still significant clinical barriers, preventing more effective cancer therapy and better clinical outcomes [1]. For example, in ER(+) breast cancer, nearly 30% to 50% of these patients develop resistance to endocrine-based therapies, such as tamoxifen [2–4]. Tamoxifen-resistance reveals itself clinically as tumor recurrence or systemic metastasis, leading to advanced disease states and pre-mature patient deaths [5]. Thus, endocrine-therapy resistance is a major determinant that significantly reduces the effectiveness of breast cancer therapy. As such, more mechanistic studies are needed to understand the molecular basis of tamoxifen-resistance at the molecular level [6, 7]. Importantly, once these key drivers of the tamoxifen-resistance phenotype are identified they may also serve as therapeutic targets and predictive biomarkers to identify high-risk patients, before they become tamoxifen-resistant [8, 9].

Here, using a combination of proteomics analysis and metabolic phenotyping, we identified enhanced mitochondrial function and oxidative stress, as key drivers of tamoxifen-resistance [10, 11]. In this context, we showed that NQO1 and GCLC, were sufficient to genetically confer tamoxifen-resistance in otherwise tamoxifen-sensitive MCF-7 cells, by enhancing their mitochondrial function [12, 13]. Thus, NQO1 and GCLC may be i) new prognostic biomarkers, ii) novel therapeutic targets and iii) companion diagnostics, for predicting and overcoming tamoxifen-resistance in different subsets of ER(+) breast cancer patients [14, 15].

## RESULTS

### Characterization of the metabolic phenotype of tamoxifen-resistant MCF-7 cell lines

In order to dissect the molecular basis of tamoxifen-resistance that occurs during endocrine-based breast cancer therapy, we used tamoxifen-resistant MCF-7 cell lines, such TAMR. The TAMR cell line is serially passaged in the presence of tamoxifen to maintain its resistance phenotype.

**Figure 1:**
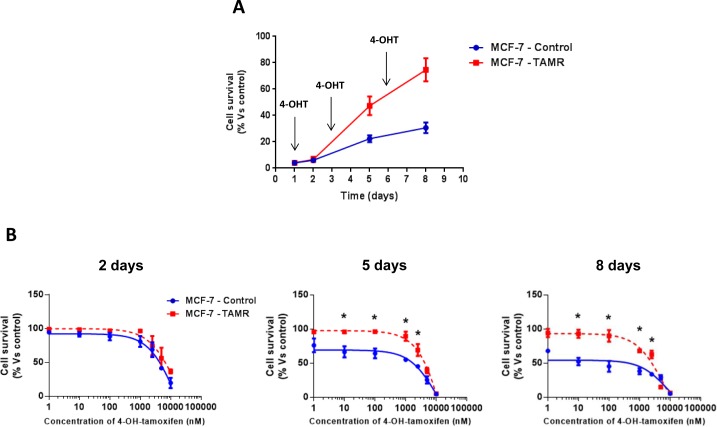
Validation of tamoxifen-resistance in TAMR cells **A**. Growth curves of MCF-7-control (WT) and MCF-7-TAMR cells in the presence of 4-OH-Tamoxifen (4-OHT) 100nM. The arrows indicate the day that fresh growth media containing 4-OHT was added to the cell cultures. **B**. Growth responses of MCF-7-control and MCF-7-TAMR cells to increasing concentrations of 4-OHT (0.1 nM-10 μM) on day 2, 5 and 8 from initial treatment. The results for the graphs are expressed as the mean (+/− SD) of six wells repeated three times. * *p* < 0.01.

First, we validated the tamoxifen-resistance phenotype of TAMR cells. Importantly, TAMR cells behaved in a tamoxifen-resistant manner and continued to grow, despite the presence of increasing concentrations of tamoxifen [16] (Figure 1).

**Figure 2:**
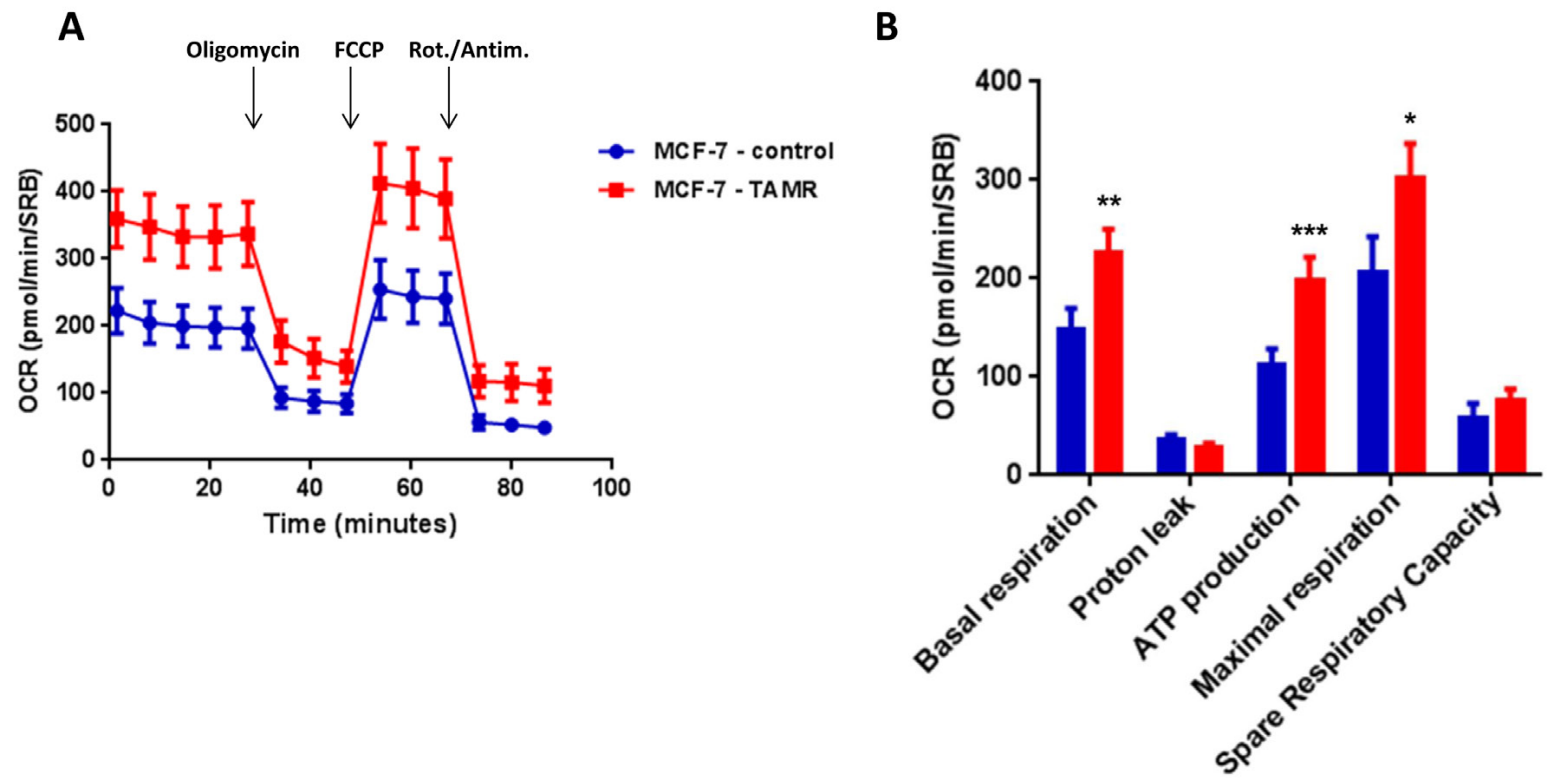
TAMR cells show a significant increase in mitochondrial oxygen consumption and mitochondrial ATP production The Seahorse XF96 analyzer was employed to determine the mitochondrial function of MCF-7-control cells and MCF-7-TAMR after 48 hours. **A**. A representative line graph of 3 independent experiments is shown. **B**. Respiration (basal and maximal), as well as ATP levels, were significantly increased.* *p* < 0.05; ** *p* < 0.005; *** *p* < 0.0005.

**Figure 3:**
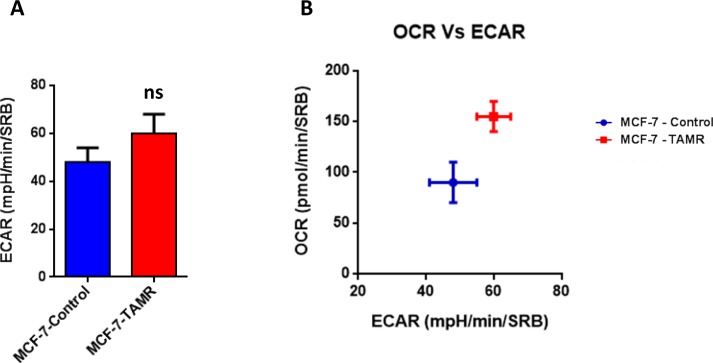
TAMR cells are more energetically active, but do not show any increases in their glycolytic rate **A**. The Seahorse XF96 analyzer was employed to determine the status of extracellular acidification rate (ECAR) in MCF-7-control and MCF-7-TAMR cells after 48 hours. A bar graph of 3 independent experiments is shown. ns = not significant. **B**. The plot of OCR *versus* ECAR shows that MCF-7-TAMR cells shift from a moderate quiescent state to a high energetic state.

**Figure 4:**
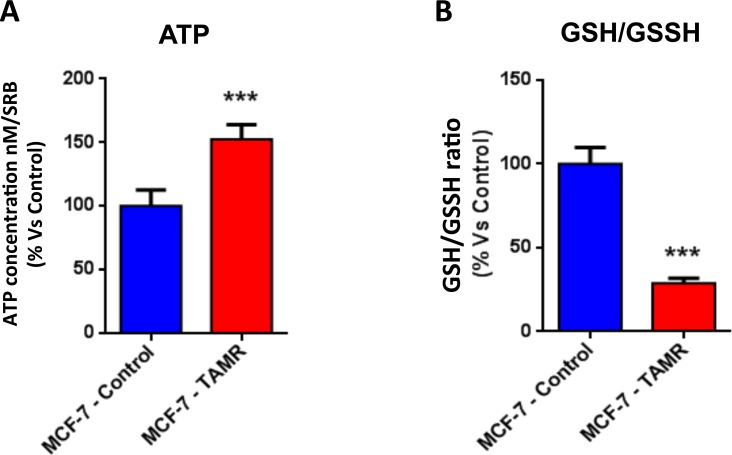
The metabolic phenotype of TAMR cells is characterized by increased steady-state levels of ATP and decreased levels of reduced glutathione **A**. ATP levels were evaluated with the Celltiter-Glo™ luminescent assay kit, after 24 hours of incubation at 37°C. **B**. The reduced/oxidized glutathione ratio, was evaluated with the GSH/GSSG-Glo™ Assay kit, after 24 hours of incubation at 37°C. Both ATP and glutathion levels were normalized by protein content (SRB) and cell number. *** *p* < 0.0005.

Then, TAMR cells were subjected to metabolic phenotyping in order to establish their behavior. For this purpose, we employed the Seahorse XF96 Analyzer to measure metabolic flux [17, 18]. Interestingly, Figure 2 illustrates that TAMR cells show an enhanced metabolic phenotype, with significant increases in oxidative mitochondrial metabolism and ATP production, as well as increased basal and maximal respiratory capacity. However, no significant increases in glycolytic rates were observed (Figure 3). This observed increase in ATP production was independently validated using a second independent biochemical assay (Figure 4A). Consistent with the idea that increased mitochondrial metabolism should lead to oxidative stress, we also found that the steady-state levels of reduced glutathione were significantly depleted (Figure 4B). Importantly, enhanced oxidative mitochondrial metabolism was also observed in a second independently-derived tamoxifen-resistant MCF-7 cell line, known as TAMR2 (Supplementary Figure 1).

**Figure 5:**
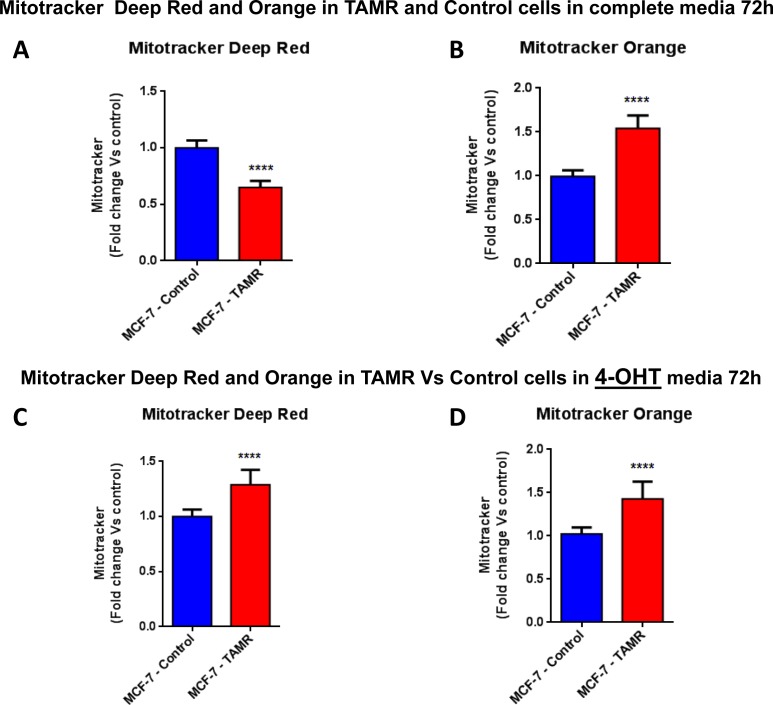
Mitochondrial biogenesis and membrane potential are increased in TAMR cells, in the presence of tamoxifen FACS analysis was carried out on MCF-7-control and MCF-7-TAMR cells after 72 hours. **A**.-**B**. In MCF-7-TAMR, the mitochondrial mass was reduced (MitoTracker Deep-Red), but an increased mitochondrial membrane potential (MitoTracker Orange) was observed after 72 hours of incubation, in growth media 4-OHT free. **C**.-**D**. The mitochondrial mass (MitoTracker Deep-Red) and mitochondrial membrane potential (MitoTracker Orange) were increased after 48 hours of incubation, in growth media containing 4-OHT. **** *p* < 0.00001.

We hypothesized that the observed increase in oxygen consumption and ATP production might occur *via* an increased capacity for mitochondrial biogenesis. In direct support of our hypothesis, TAMR cells showed a significant increase in both mitochondrial mass and mitochondrial membrane potential (Figure 5), as observed by FACS analysis using MitoTracker vital dyes as probes (Deep Red and Orange) [19, 20]. However, this phenotype was strictly dependent on the presence of tamoxifen in the tissue culture media.

### Proteomics Analysis of TAMR Cells: NQO1 and GCLC are strongly up-regulated

To begin to dissect the molecular basis of this enhanced metabolic phenotype, TAMR cells were next subjected to unbiased proteomics analysis [21]. This would allow us to identify potential therapeutic targets that might confer tamoxifen-resistance. These results are summarized in Table 1. We focused on the top 20 proteins that were over-expressed in TAMR cells, relative to matched parental MCF-7 cells.

**Table 1:** List of top 20 proteins up-regulated in tamoxifen-resistant MCF7 cells (MCF-7-TAMR)

Symbol	Description	Fold-Increase (Up-regulation)
GPATCH8	G patch domain-containing protein 8	Infinity
SRRM2	Serine/arginine repetitive matrix protein 2	Infinity
HSD17B4	Peroxisomal multifunctional enzyme type 2	286.23
AKR1C2	Aldo-keto reductase family 1 member C2	182.79
NQO1	NAD(P)H dehydrogenase [quinone] 1	74.05
BCAS1	Breast carcinoma amplified sequence 1	54.13
CTNND1	Catenin delta-1	35.23
GCLC	Glutamate-cysteine ligase	29.32
BMP7	Bone morphogenetic protein 7	28.74
PI4KA	Phosphatidylinositol 4-kinase alpha	25.59
IDH2	Isocitrate dehydrogenase [NADP], mitochondrial	22.95
ACAA2	3-ketoacyl-CoA thiolase, mitochondrial	22.10
GPCPD1	Glycerophosphocholine phosphodiesterase	18.01
UGDH	UDP-glucose 6-dehydrogenase	11.31
SMC1A	Structural maintenance of chromosomes protein 1A	11.03
ATP12A	Potassium-transporting ATPase alpha chain 2	10.89
ATP1B1	Sodium/potassium-transporting ATPase subunit beta-1	10.61
STAU1	staufen double-stranded RNA binding protein 1	10.03
AHNAK	Neuroblast differentiation-associated protein	9.91
S100P	Protein S100-P	9.76

Note that several of the up-regulated proteins are metabolic enzymes, as predicted. In this data set, we also noticed that the BCAS1 protein was upregulated by > 50-fold. Importantly, BCAS1 has been previously implicated in conferring tamoxifen-resistance. Thus, we used BCAS1 as a positive control in some of our experiments.

**Figure 6:**
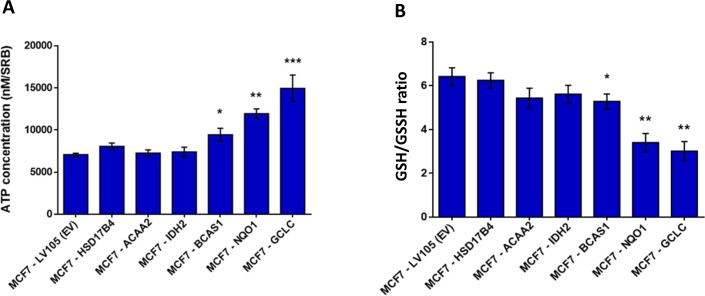
Energetic screening in MCF-7 cells transduced with different cDNAs encoding a subset of proteins up-regulated in TAMR cells **A**. ATP levels were evaluated with the Celltiter-Glo™ luminescent assay kit, after 24 hours of incubation at 37°C. **B**. The reduced/oxidized glutathione ratio was evaluated with the GSH/GSSG-Glo™ Assay kit, after 24 hours of incubation at 37°C. Both ATP and glutathion levels were normalized by protein content (SRB) and cell number. * *p* < 0.05; ** *p* < 0.005.

To begin to test our hypothesis that these genes might confer tamoxifen-resistance, we transduced a panel of six candidates into MCF-7 cells (NQO1, GCLC, ACAA2, IDH2, HSD17B4 and BCAS1) and then evaluated their metabolic phenotype. As a more efficient screening method, we chose to measure ATP and glutathione levels, to ask which of these candidates would be sufficient to mimic the metabolic phenotype of TAMR cells. As predicted, this screening method was successful and three out of the six candidates (BCAS1, NQO1 and GCLC) actually increased ATP and decreased reduced glutathione (Figure 6). Since NQO1 and GCLC conferred the largest changes in ATP and reduced glutathione, we decided to focus on these two metabolic enzymes as potential mediators of tamoxifen-resistance. NQO1 and GCLC were increased by 74-fold and by 29-fold in TAMR cells, respectively, relative to parental MCF-7 cells (Table 1).

### NQO1 and GCLC are sufficient to confer tamoxifen-resistance and “boost” mitochondrial metabolism

Based on our proteomics analysis and screening approach, NQO1 and GCLC emerged as the most promising potential targets. Thus, we subjected these MCF-7-NQO1 and MCF-7-GCLC cell lines to further characterization. We performed additional mechanistic studies, to determine if they truly mimic the tamoxifen-resistance behavior and metabolic phenotype of TAMR cells.

**Figure 7:**
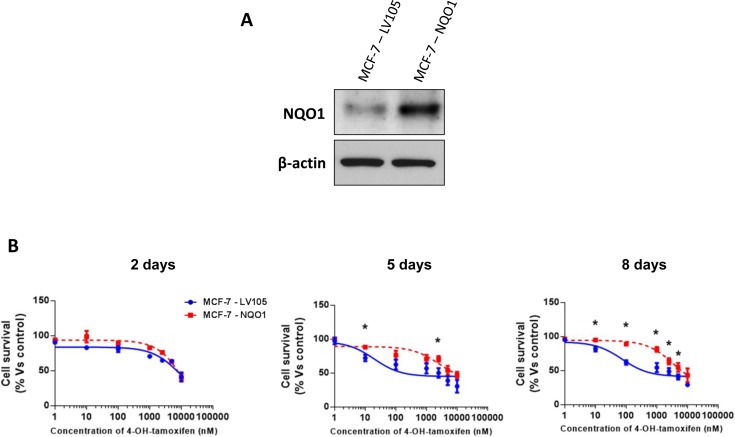
MCF-7 cells over-expressing NQO1 are tamoxifen-resistant **A**. MCF-7 cells were stably transduced with the cDNA encoding NQO1 or the empty vector plasmid (LV105-puro), using a lentiviral vector approach. Total cell proteins were isolated from transfected MCF-7 cells and analyzed by immunoblotting to confirm NQO1 expression. NQO1 antibody (#HPA007308 Sigma-Aldrich) was used with a dilution 1:500 in 5% of BSA. The expression of β-actin (#A2228 Sigma-Aldrich) was also assessed to ensure equal protein loading. **B**. Growth responses of MCF-7-LV105 and MCF-7-NQO1 cells to increasing concentrations of 4-OHT (0.1 nM-10μM) on day 2, 5 and 8 from the initial treatment. The graphs are expressed as the mean (+/− SD) of six wells repeated three times. * *p* < 0.01.

**Figure 8:**
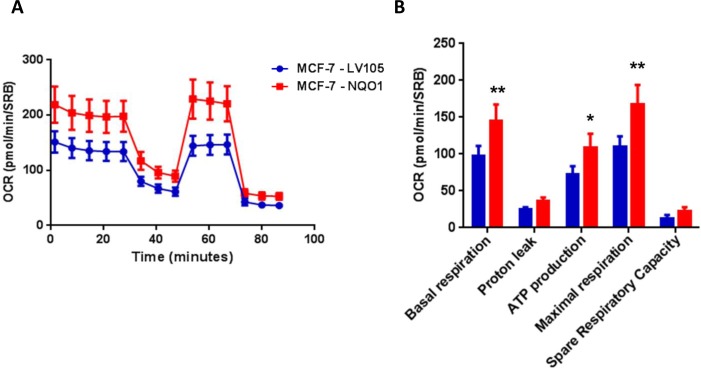
NQO1 expressing MCF7 cells show a significant increase in mitochondrial oxygen consumption and mitochondrial ATP production The Seahorse XF96 analyzer was employed to determine the status of mitochondrial function in MCF-7-LV105 cells (empty vector) and MCF-7-NQO1 cells after 48 hours. **A**. A representative line graph of 3 independent experiments is shown. **B**. Respiration (basal and maximal), as well as ATP levels, were significantly increased. * *p* < 0.05; ** *p* < 0.005.

**Figure 9:**
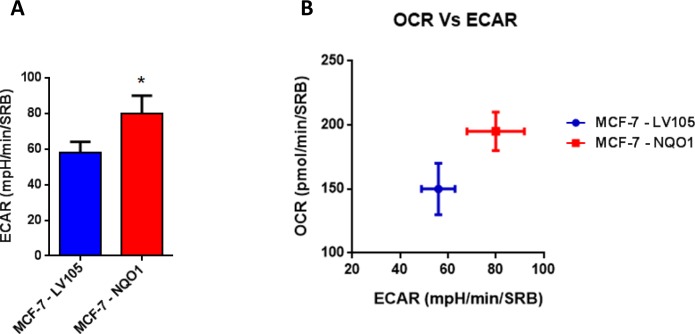
NQO1 expressing MCF7 cells are more energetically active, and show a minor increase in their glycolytic rate **A**. The Seahorse XF96 analyzer was employed to determine the status of extracellular acidification rate (ECAR) in MCF-7-LV105 and MCF-7- NQO1 cells after 48 hours. A bar graph of 3 independent experiments is shown. **B**. The plot of OCR *versus* ECAR shows that MCF-7-TAMR cells shift from moderate quiescent state to a high energetic state. * *p* < 0.05.

**Figure 10:**
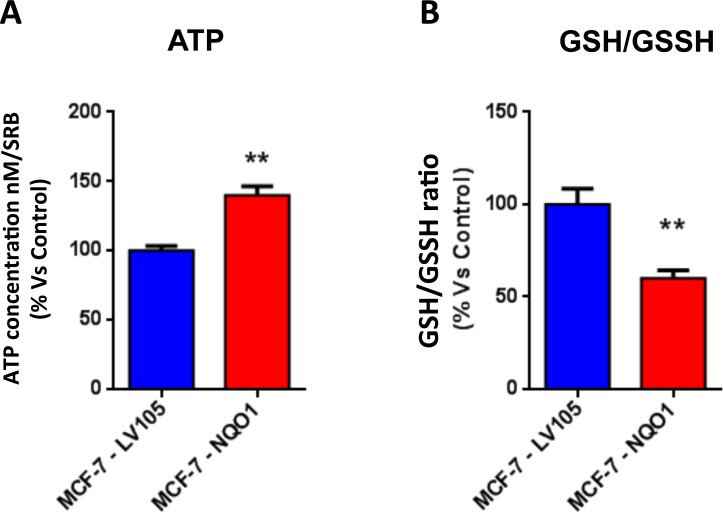
The metabolic phenotype of NQO1 expressing MCF7 cells is characterized by increased steady-state levels of ATP and decreased levels of reduced glutathione **A**. ATP concentration levels, evaluate with Celltiter-Glo™ luminescent assay kit, after 24 hours of incubation at 37°C. **B**. Reduced/oxidized glutathione ratio, evaluate with GSH/GSSG-Glo™ Assay kit, after 24 hours of incubation at 37°C. Both types of experiments were standardized by protein content (SRB) and cell number. ** *p* < 0.005.

**Figure 11:**
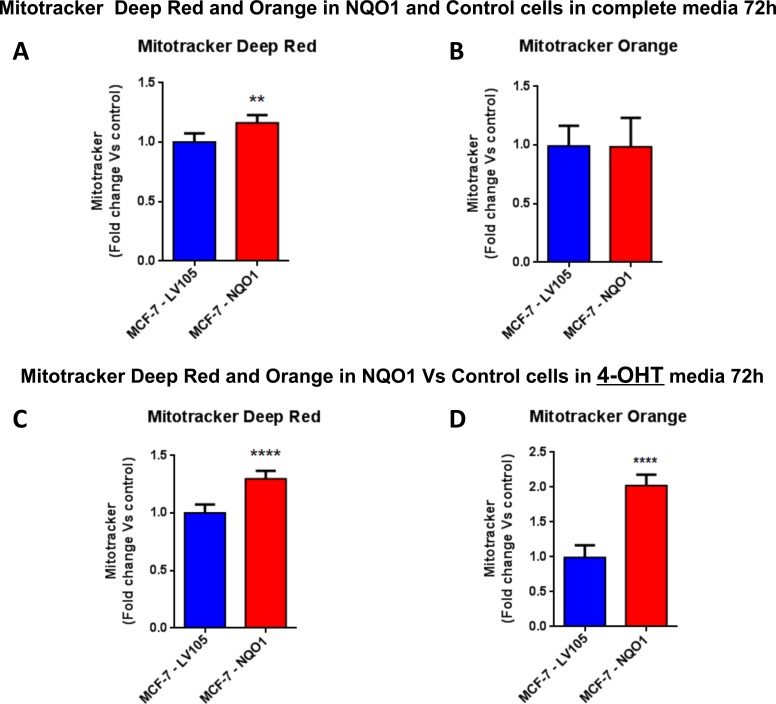
Mitochondrial biogenesis and membrane potential are increased in NQO1 expressing MCF7 cells, in the presence of tamoxifen FACS analysis was carried out on MCF-7-LV105 (empty vector) and MCF-7-NQO1 after 72 hours. **A**.-**B**. In MCF-7-NQO1, the mitochondrial mass was increased (MitoTracker Deep-Red), but not significant changes in mitochondrial membrane potential (MitoTracker Orange) were observed after 72 hours of incubation, in growth media 4-OHT free. **C**.-**D**. The mitochondrial mass (MitoTracker Deep-Red) and mitochondrial membrane potential (MitoTracker Orange) were increased after 48 hours of incubation, in growth media containing 4-OHT. ** *p* < 0.001; **** *p* < 0.00001.

Remarkably, recombinant over-expression of NQO1 was indeed sufficient to functionally confer tamoxifen-resistance (Figure 7). Importantly, empty-vector control MCF-7 cell lines tested in parallel were tamoxifen-sensitive. Similarly, NQO1 over-expression significantly increased oxidative mitochondrial metabolism, with increased levels of ATP production and decreased levels of reduced glutathione (Figures 8, 9 and 10). Finally, mitochondrial biogenesis and mitochondrial membrane potential were also increased in MCF-7-NQO1 cells (Figure 11).

**Figure 12:**
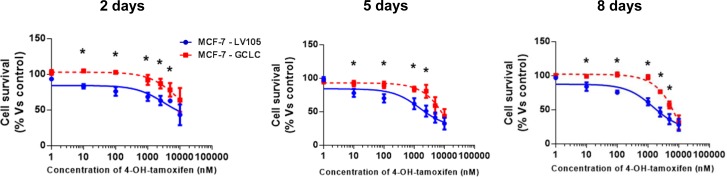
MCF-7 cells harboring GCLC show tamoxifen-resistance Growth responses of MCF-7-LV105 (empty vector) and MCF-7-GCLC cells to increasing concentrations of 4-OHT (0.1 nM-10μM) on day 2, 5 and 8 from initial treatment. The results for the graphs are expressed as the mean (+/−SD) of six wells repeated three times. * *p* < 0.01.

**Figure 13:**
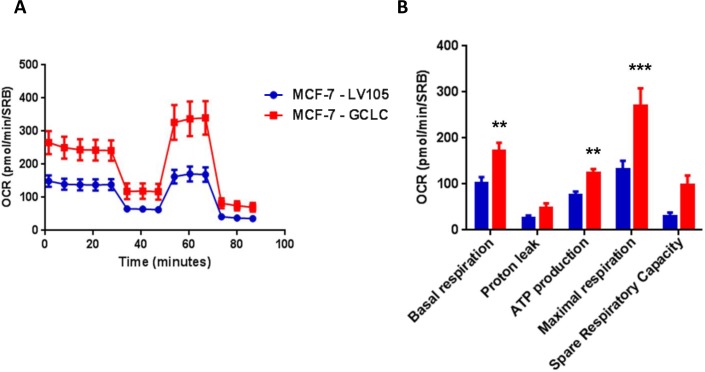
MCF7 cells harboring GCLC show a significant increase in mitochondrial oxygen consumption and mitochondrial ATP production The Seahorse XF96 analyzer was employed to determine the mitochondrial function of MCF-7-LV105 (empty vector) and MCF-7-GCLC cells after 48 hours. **A**. A representative line graph of 3 independent experiments is shown. **B**. Respiration (basal and maximal), as well as ATP levels, were significantly increased.** *p* < 0.005; *** *p* < 0.0005.

**Figure 14:**
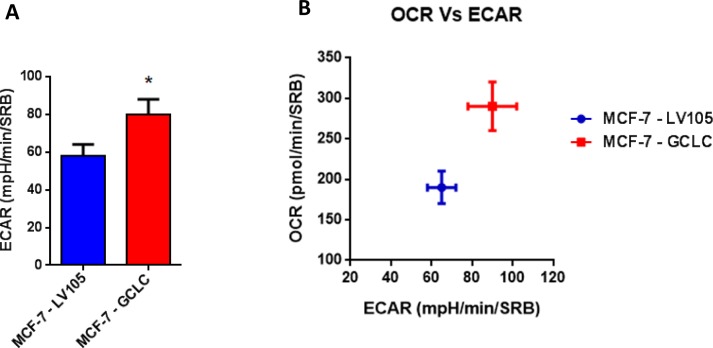
MCF7 cells harboring GCLC are more energetically active, and show a minor increase in their glycolytic rate The Seahorse XF96 analyzer was employed to determine the status of extracellular acidification rate (ECAR) in MCF-7-LV105 (empty vector) and MCF-7-GCLC cells after 48 hours. A bar graph of 3 independent experiments is shown. **B**. The plot of OCR *versus* ECAR shows that MCF-7-TAMR cells shift from moderate quiescent state to a high energetic state. * *p* < 0.05.

**Figure 15:**
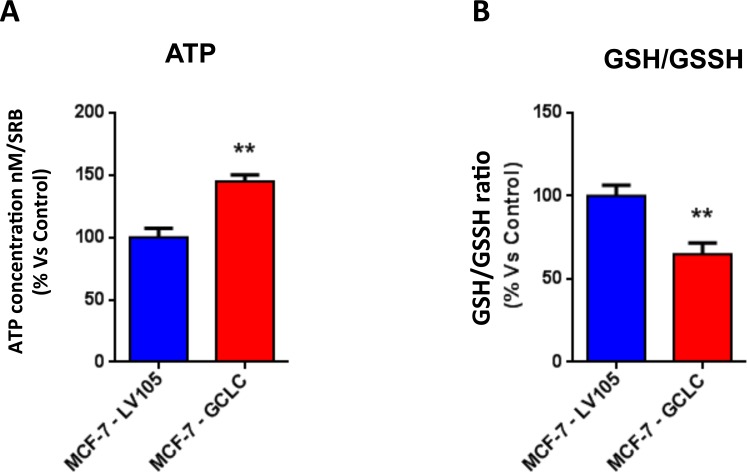
MCF7 cells harboring GCLC are characterized by increased steady-state levels of ATP and decreased levels of reduced glutathione **A**. ATP levels evaluated with the Celltiter-Glo™ luminescent assay kit, after 24 hours of incubation at 37°C. **B**. The reduced/oxidized glutathione ratio was evaluated with the GSH/GSSG-Glo™ Assay kit, after 24 hours of incubation at 37°C. Both assays were normalized by protein content (SRB) and cell number. ** *p* < 0.005.

**Figure 16:**
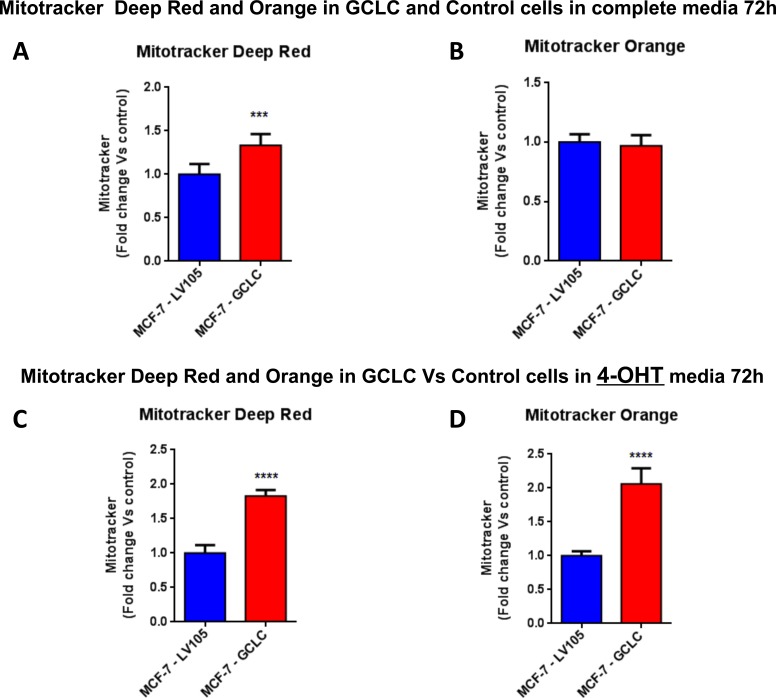
Mitochondrial biogenesis and membrane potential are increased in MCF7 cells harboring GCLC, in the presence of tamoxifen FACS analysis was carried out on MCF-7-LV105 (empty vector) and MCF-7-GCLC after 72 hours. **A**.-**B**. In MCF-7-GCLC, the mitochondrial mass was increased (MitoTracker Deep-Red), but no significant change in mitochondrial membrane potential (MitoTracker Orange) was observed after 72 hours of incubation, in growth media 4-OHT free. **C**.-**D**. Both the mitochondrial mass (MitoTracker Deep-Red) and mitochondrial membrane potential (MitoTracker Orange) were highly increased after 48 hours of incubation, in growth media containing 4-OHT. *** *p* < 0.0001; **** *p* < 0.00001.

Importantly, virtually identical results were also obtained with GCLC. MCF-7 cells transduced with the cDNA of GCLC were i) tamoxifen-resistant (Figure 12) and ii) showed a significant increase in mitochondrial oxygen consumption (Figure 13 and 14). Moreover, MCF-7-GCLC cells had enhanced ATP production and showed decreased reduced glutathione (Figure 15), with increased mitochondrial biogenesis (Figure 16).

In accordance with the above functional data, a number of mitochondrial and/or other metabolism-related proteins were highly up-regulated, as seen by proteomics analysis, in both NQO1 and GCLC transfected MCF-7 cells (Tables 2 and 3). These up-regulated mitochondrial proteins included ABAT, AIFM1, ATPIF1, GSR, MRPS22 and PRDX5, among others.

**Table 2:** List of top 27 proteins up-regulated in MCF-7-NQO1 cells

Symbol	Description	Fold-Increase (Up-regulation)
		
MED13	Mediator of RNA polymerase II transcription subunit 13	Infinity
**PRDX5**	Peroxiredoxin-5, mitochondrial	16.99
HSPA4L	Heat shock 70 kDa protein 4L	16.29
MYO18B	Unconventional myosin-XVIIIb	10.10
RAB21	Ras-related protein Rab-21	8.74
ADK	Adenosine kinase	7.60
SQSTM1-ALK	Tyrosine-protein kinase receptor	3.97
PAMR1	Inactive serine protease PAMR1	3.93
LARP4	La-related protein 4	3.23
BAG3	BAG family molecular chaperone regulator 3	3.21
UCHL5	Ubiquitin carboxyl-terminal hydrolase L5	2.96
KATNAL2	Katanin p60 ATPase-containing subunit A-like 2	2.96
**SLC25A3**	Phosphate carrier protein, mitochondrial	2.91
**PKM2**	Pyruvate kinase	2.82
FPGT	FPGT protein	2.81
RPS4Y1	40S ribosomal protein S4, Y isoform 1	2.79
**MRPS22**	28S ribosomal protein S22, mitochondrial	2.78
PSMB	Proteasome subunit beta type	2.77
PRKRA	Interferon-inducible ds-RNA-dependent protein kinase	2.70
CLEC4C	C-type lectin domain family 4 member C	2.66
YWHAE	14-3-3 protein epsilon	2.63
KRT14	Keratin, type I cytoskeletal 14	2.46
PAMR1	Inactive serine protease PAMR1	2.39
CEP110	Centrosomal protein 110kDa	2.38
CCT7	Chaperonin containing TCP1, subunit 7 (Eta) variant	2.37
CTSD	Cathepsin D	2.33
**GSR**	Glutathione reductase, mitochondrial	2.31

Note that the following 5 mitochondrial and/or metabolism-related enzymes are up-regulated in MCF-7-NQO1 cells (PRDX5, SLC25A3, PKM2, MRPS22, GSR) and are highlighted in BOLD.

**Table 3:** List of top 29 proteins up-regulated in MCF-7-GCLC cells

Symbol	Description	Fold-Increase (Up-regulation)
CPSF6	Cleavage and polyadenylation-specificity factor subunit 6	Infinity
GSPT1	Eukaryotic peptide chain release factor GTP-binding subunit ERF3A	5,0127.30
**AIFM1**	Apoptosis-inducing factor 1, mitochondrial	111.08
DYNC1H1	Cytoplasmic dynein 1 heavy chain 1	109.20
VIM	Vimentin	55.27
TTN	Titin	33.27
**ATPIF1**	ATPase inhibitor, mitochondrial	30.89
CYP21A2	Steroid 21-hydroxylase	23.49
SERPINH1	Serpin H1	22.11
KRT4	Keratin, type II cytoskeletal 4	20.88
NOL3	Nucleolar protein 3	20.15
BAG3	BCL2-associated athanogene 3	18.40
CTSD	Cathepsin D	17.87
HNRNPA2B1	Heterogeneous nuclear ribonucleoproteins A2/B1	17.87
ZCCHC11	Terminal uridylyltransferase 4	16.47
2ATP12A	Potassium-transporting ATPase alpha chain	16.18
YWHAZ	14-3-3 protein zeta/delta (Fragment)	15.70
L27a	Ribosomal protein L27a	14.84
STUB1	E3 ubiquitin-protein ligase CHIP	14.55
NCL	Nucleolin	11.61
**G6PD**	Glucose-6-phosphate 1-dehydrogenase	11.25
HSPA4L	Heat shock 70 kDa protein 4L	10.40
FASN	FASN protein	10.18
**PGK2**	Phosphoglycerate kinase 2	10.12
UGP2	UTP--glucose-1-phosphate uridylyltransferase	9.89
HMGN4	High mobility group nucleosome-binding domain-containing protein 4	9.06
DCXR	L-xylulose reductase	9.00
CTNNA2	Catenin alpha-2	8.56
**ABAT**	4-aminobutyrate aminotransferase, mitochondrial	7.32

Note that the following 5 mitochondrial and/or metabolism-related enzymes are up-regulated in MCF-7-GCLC

In summary, the NQO1 and GCLC genes are both indeed sufficient to confer tamoxifen-resistance and to “boost” mitochondrial metabolism. Conversely, we also observed that treatment of MCF-7-TAMR cells with dicoumarol, a known NQO1 inhibitor, reverses their tamoxifen-resistance phenotype (Supplementary Figures 2 and 3). Thus, pharmacological inhibition of NQO1 and GCLC may be new therapeutic strategies for overcoming tamoxifen-resistance in breast cancer patients.

### Overview of proteins and pathways identified as differentially regulated in the lysates of TAMR, NQO1 and GCLC cells relative to control MCF-7 cells and MCF-7-LV105 (empty vector), by ingenuity pathway analysis

**Figure 17:**
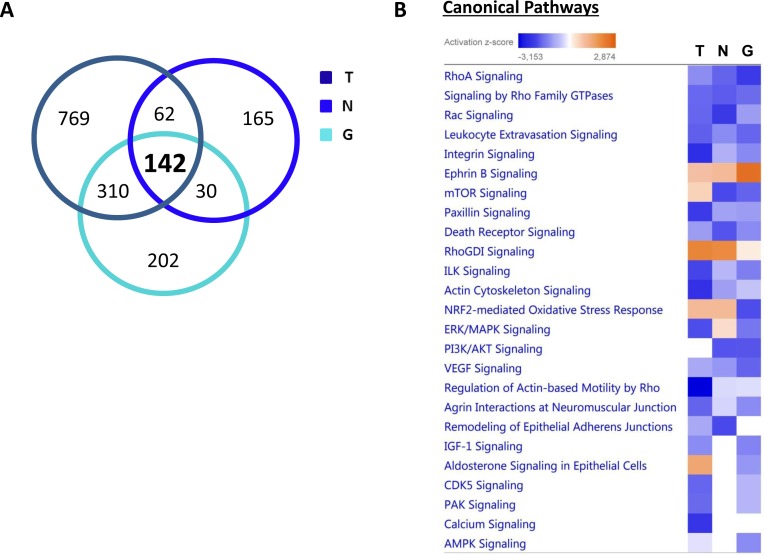
Overview of proteins and pathways identified as differentially regulated in the lysates of TAMR, NQO1 and GCLC cells relative to control MCF7 cells, by Ingenuity Pathway Analysis **A**. Venn diagram. Overlap of differentially regulated proteins identified in MCF-7-TAMR, MCF-7-NQO1 and MCF-7-GCLC, compared with their proper controls. Of all the proteins identified by quantitative proteomics, 142 were proteins that the expression of which was found to be altered in both treatments, compared to control. **B**. Canonical pathways identified or predicted as altered in MCF-7-TAMR, MCF-7-NQO1 and MCF-7-GCLC, relative to control. A positive z score is indicated in orange and points towards an activation of the pathway, and a negative z score, in blue, indicates an inhibition of the pathway. (T = MCF-7-TAMR *vs*. MCF-7-control; N = MCF-7-NQO1 *vs*. MCF-7-LV105; G = MCF-7-GCLC *vs*. MCF-7-LV105).

**Figure 18:**
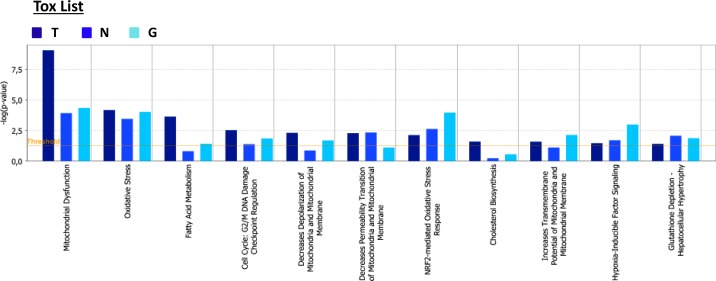
Tox list of pathways identified as differentially regulated in the lysates of TAMR, NQO1 and GCLC cells relative to control MCF7 cells, by Ingenuity Pathway Analysis Toxicity effects of differentially expressed proteins in MCF-7-TAMR, MCF-7-NQO1 and MCF-7-GCLC compared to control cells. Ingenuity Pathway Analysis showed toxicity functions significantly enriched by the proteins differentially expressed in the comparison analysis (*p* < 0.05). In the Bar chart, the p value for each pathway is indicated by the bar and is expressed as -1 times the log of the p value. (T = MCF-7-TAMR *Vs* MCF-7-control; N = MCF-7-NQO1 *Vs* MCF-7-LV105 (empty vector); G = MCF-7-GCLC *Vs* MCF-7-LV105 (empty vector)).

From all the proteomics data sets, the differentially expressed proteins were subjected to Ingenuity Pathway Analysis (IPA) to determine possible alterations in canonical pathways and toxicity functions. IPA was able to analyze 1,283 proteins in MCF-7-TAMR, 399 proteins in MCF-7-NQO1 and 684 proteins in MCF-7-GCLC cells, as compared with their respective controls. This analysis revealed that 142 proteins were differentially regulated in all 3 different cell lines (Figure 17A). A higher presence of antioxidant response proteins was further confirmed using IPA, which revealed the Nrf2-mediated antioxidant response to be one of the top canonical pathways affected. Similarly, TAMR, NQO1 and GCLC cells all showed very similar regulation of numerous cellular pathways (Figure 17B). Importantly, the Heat-Map shows how the different cell lines behave almost in the same way, emphasizing their similarities also in the regulation of numerous cancer-related signaling pathways. Thus, our proteomics analysis clearly detects alterations in mitochondrial function, the Nrf-2-mediated oxidative stress response and Hypoxia-Inducible factor (HIF) signaling, all mediated by NQO1 and GCLC as compared with MCF-7-TAMR cells (Figure 18).

### Bioinformatic validation of the clinical relevance of NQO1 and GCLC in human breast cancer patients, in various ER(+) epithelial sub-types

To assess the possible clinical relevance of NQO1 and GCLC, we determined if their mRNA levels showed any prognostic value in human breast cancer patient cohorts, with long-term follow-up (nearly 20 years). The prognostic value of NQO2 was also evaluated in parallel, for comparison purposes. We restricted our analysis to ER(+) patients that received endocrine therapy (mostly tamoxifen), but not any form of chemotherapy.

**Figure 19:**
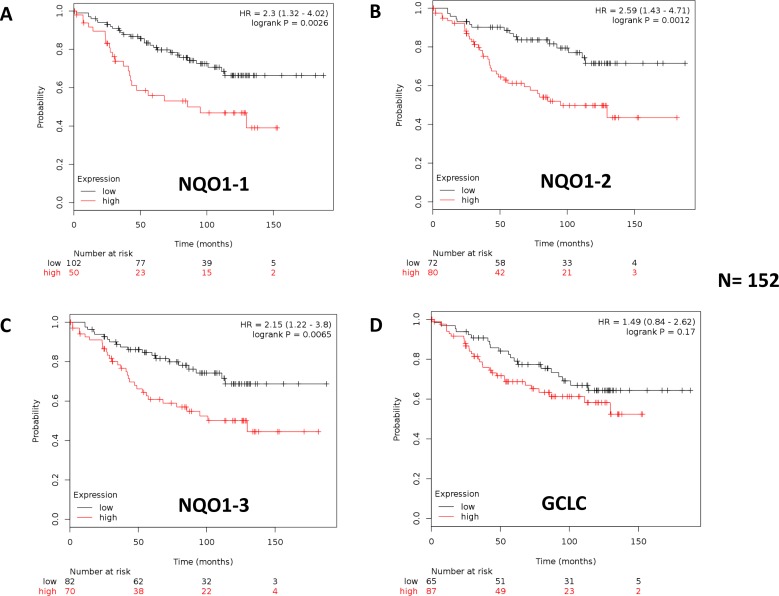
Kaplan-Meier (K-M) analysis of the prognostic value of NQO1 and GCLC in ER(+) breast cancer patients receiving endocrine therapy: Luminal A subgroup Results of recurrence-free survival analysis (RFS) are shown, over a > 15-year period of follow-up, for NQO1 (3 independent probes; **A**.-**C**., and GCLC **D**., for ER(+) breast cancer patients (N = 152) with the luminal A sub-type. These patients were lymph-node-positive (LN(+)) and received endocrine therapy (mostly tamoxifen), but not chemotherapy. Note that higher levels of NQO1 mRNA species **A**.-**C**. are significantly associated with tumor recurrence. See the *Methods section* for how the analysis was carried out. Similar results were obtained in other ER(+) sub-types of breast cancer, and are included as Supplementary Figures 4 and 5.

These results are summarized in Table 4, and in Supplementary Tables S1 and S2. Corresponding Kaplan-Meier (K-M) analysis curves are included in Figure 19, and as Supplementary Figures 4 and 5 (panels A-D).

**Table 4:** Prognostic Value of NQO1, NQO2 and GCLC in a High-Risk Subgroup of ER(+) Patients: Luminal A/LN(+)

Symbol	Probe Number	Hazard Ratio (HR)	P-Value (LogRank)
NQO1-1	201467_s_at	2.30	0.0026
NQO1-2	201468_s_at	2.59	0.0012
NQO1-3	210519_s_at	2.15	0.0065
NQO2	203814_s_at	1.52	0.14
GCLC	202923_s_at	1.49	0.17

Recurrence-free survival (RFS) in a high-risk population of breast cancer patients:

ER(+)/Luminal A/LN(+)/EndocrineTx (mostly Tamoxifen)/**N=152 patients**.

Note that high mRNA expression levels of all three distinct NQO1 probes showed an association with reduced relapse-free survival (RFS, i.e., higher tumor recurrence). More specifically, NQO1 had prognostic value in Luminal A patients with lymph node metastasis (LN(+)), as well in Luminal B patients, and in the total ER(+) patient population. GCLC only showed prognostic value in the Luminal B sub-population of ER(+) breast cancer patients. In contrast, NQO2 mRNA levels did not show any prognostic value in any of the ER(+) patient groups examined (Table 4; Supplementary Tables S1 and S2).

Table 4: **Prognostic value of NQO1, NQO2 and GCLC in a high-risk subgroup of ER(+) patients: Luminal A/LN(+)**

Finally, since high levels of NQO1 mRNA were associated with disease progression in patients that received endocrine therapy, this is indicative of a clinical association with endocrine therapy-resistance. Thus, elevated levels of NQO1 expression could be used to identify high-risk ER(+) breast cancer patients, that might benefit from treatment with novel NQO1 inhibitors.

## DISCUSSION

**Figure 20:**
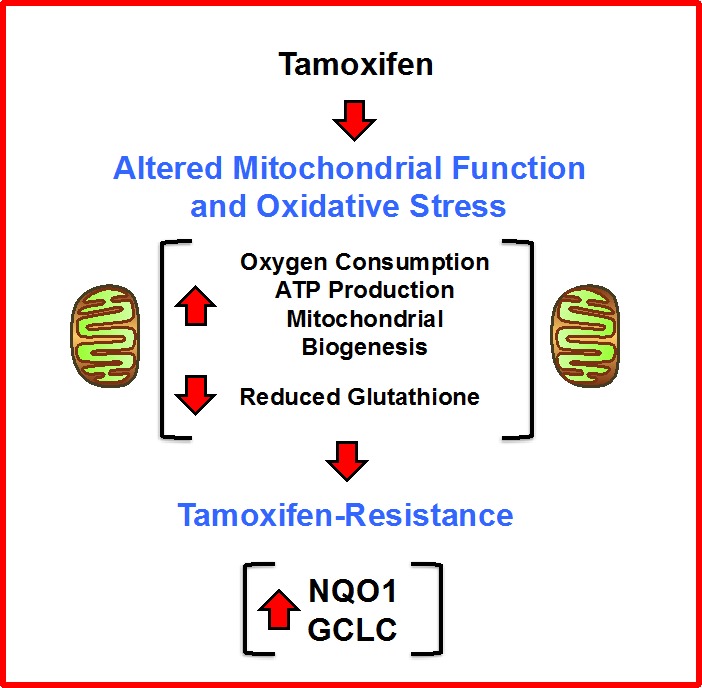
Enhanced mitochondrial metabolism: A new mechanism for driving tamoxifen-resistance A schematic diagram is shown high-lighting our observations that increased mitochondrial oxygen-consumption and ATP-production, as well as increased mitochondrial biogenesis and oxidative stress, all contribute to the metabolic phenotype of tamoxifen-resistant breast cancer cells. In this process, the over-expression of NQO1 and GCLC appear to be key drivers of this phenotype.

In this report, we set out to identify new metabolic drivers of tamoxifen-resistance, by combining proteomics analysis with metabolic phenotyping [22, 23]. Use of this proteomics-metabolomics approach allowed us to identify enhanced mitochondrial metabolism as a key characteristic of TAMR cells, that was characterized by i) increased oxygen consumption, ii) increased ATP production, and iii) augmented mitochondrial biogenesis, as well as increased oxidative stress, as revealed by iv) decreased levels of reduced glutathione levels (Figure 20). Thus, we focused the interpretation of our proteomics results on key metabolic enzymes that were dramatically increased in tamoxifen-resistant cells, such as NQO1 and GCLC. To determine if the over-expression of NQO1 and/or GCLC could autonomously confer the tamoxifen-resistance phenotype, we transduced MCF-7 cells with the cDNAs encoding these proteins, using a lenti-viral vector approach. Remarkably, NQO1 and GCLC lenti-viral transduction was indeed sufficient to induce tamoxifen-resistance, as well as the metabolically-enhanced mitochondrial phenotype.

Thus, increased mitochondrial function may help drive tamoxifen-resistance in human breast cancer cells (Figure 20). This key finding has important mechanistic implications for understanding the molecular basis of tamoxifen-resistance. Furthermore, the over-expression of NQO1 mRNA species predicted tumor recurrence in high-risk ER(+) that were LN(+) and had received endocrine-therapy (mostly tamoxifen), but not chemotherapy. As such, our findings may have translational and clinical relevance for over-coming the resistance to endocrine therapy in ER(+) breast cancer patients [24].

It remains unknown exactly how NQO1 and GCLC drive an enhanced mitochondrial metabolic phenotype. The NQO1 protein product is an enzyme known as NAD(P)H dehydrogenase [quinone] 1. It is the main non-mitochondrial enzyme responsible for the reduction of CoQ10 in cells. Thus, NQO1 may “boost” mitochondrial metabolism by providing increased reduced CoQ10 species to facilitate oxidative mitochondrial metabolism [25, 26].

The GCLC protein product is an enzyme known as glutamate-cysteine ligase catalytic subunit. Glutamate-cysteine ligase is also known as gamma-glutamyl-cysteine synthetase, and is the first and rate-limiting step for glutathione synthesis. While it remains unknown how GCLC regulates mitochondrial function, its deletion in mouse liver leads to striking decreases in mitochondrial function, mitochondrial injury, loss of cellular ATP, and a marked increase in lipid peroxidation, driving steatosis and liver failure. Interestingly, treatment of these mice with oral N-acetyl-cysteine (NAC) restores mitochondrial function and prevents mortality caused by the loss of hepatocyte GSH synthesis [27, 28].

NQO1 and GCLC are normally up-regulated as part of the Nrf2-anti-oxidant response, which occurs during oxidative stress [12, 24]. Thus, the observed up-regulation of NQO1 and GCLC in TAMR cells may directly reflect that tamoxifen treatment can induce oxidative stress. How does tamoxifen induce oxidative stress? Several different mechanisms have been proposed to explain the manner in which tamoxifen induces oxidative stress in cells, including the targeting of NOS, SOD2 and ER-beta in mitochondria.

Tamoxifen also has direct effects on mitochondrial function. Tamoxifen acts both as an uncoupling agent and as a potent inhibitor of electron transport, ultimately leading to a collapse of the mitochondrial membrane potential and cellular apoptosis [29–31]. More specifically, tamoxifen inhibits electron transfer at the levels of both complex III (ubiquinol-cytochrome-c reductase) and complex IV (cytochrome-c oxidase). Similarly, it has also been reported that tamoxifen elevates mitochondrial ROS, mitochondrial lipid peroxidation and cytochrome c release, while driving the tyrosine-nitration of key mitochondrial proteins.

Therefore, our current findings could mechanistically explain why “boosting” mitochondrial metabolism may lead to tamoxifen-resistance in human breast cancer cells. If tamoxifen indeed behaves as a mitochondrial “poison”, then increased mitochondrial “power” could help to buffer against the mitochondrial toxicity effects of tamoxifen, thereby conferring tamoxifen-resistance.

## MATERIALS AND METHODS

### Materials

MCF7 cells (catalogue # HTB-22), a human breast cancer cell line, were originally obtained from the ATCC cell repository. Tamoxifen and dicoumarol were obtained commercially from Sigma-Aldrich, Inc.

### Label-free semi-quantitative proteomics analysis

Cell lysates were prepared for trypsin digestion by sequential reduction of disulphide bonds with TCEP and alkylation with MMTS. Then, the peptides were extracted and prepared for LC-MS/MS. All LC-MS/MS analyses were performed on an LTQ Orbitrap XL mass spectrometer (Thermo Scientific, San Jose, CA) coupled to an Ultimate 3000 RSLCnano system (Thermo Scientific, formerly Dionex, The Netherlands). Xcalibur raw data files acquired on the LTQ-Orbitrap XL were directly imported into Progenesis LCMS software (Waters Corp) for peak detection and alignment. Data were analyzed using the Mascot search engine. Five technical replicates were analyzed for each sample type [32].

### Ingenuity pathway analyses

Pathway and function analyses were generated using Ingenuity Pathway Analysis (IPA) (Ingenuity systems, http://www.ingenuity.com), which assists with proteomics data interpretation *via* grouping differentially expressed genes or proteins into known functions and pathways. Pathways with a z score > 1.5 were considered as significantly activated, and pathways with a z score < -1.5 were considered as significantly inhibited.

### Lentiviral gene transduction

Lentiviral plasmids, packaging cells and reagents were purchased from Genecopoeia. 48 hours after seeding, 293Ta packaging cells were transfected with lentiviral vectors encoding NQO1 or GCLC or empty vector (EX-NEG-Lv105), using Lenti-PacTM HIV Expression Packaging Kit according to the manufacturer's instructions. Two days post-transfection, lentivirus-containing culture medium was passed through a 0.45 μm filter and added to the target cells (MCF-7 cells) in the presence of 5μg/ml Polybrene. Infected cells were selected with a concentration of 1.5 μg/ml of puromycin.

### Cellular growth using the sulforhodamine B (SRB) assay

SRB (S9012, Sigma) measures total biomass by staining cellular proteins. After 2, 5 and 8 days, cells were fixed in 10% trichloroacetic acid (T9159, Sigma) for 1h at 4°C, stained with SRB (S9012, Sigma) for 15 minutes, and washed 3 times with 1% acetic acid (27225, Sigma). The incorporated die was solubilized with 10 mM Tris Base, pH 8.8 (T1503, Sigma). Absorbance was spectrophotometrically measured at 540 nm in a FluoStar Omega plate reader (BMG Labtech). Background measurements were subtracted from all values. Dicoumarol was obtained from Sigma-Aldrich, Inc.

### Seahorse XF96 metabolic flux analysis (OCR and ECAR)

Real-time oxygen consumption rates (OCR) and extracellular acidification rates (ECAR) measurements were determined using the Seahorse Extracellular Flux (XF96) analyzer (Seahorse Bioscience, MA, USA). 1 × 10^4^ cells per well were seeded into XFe-96 well cell culture plates, and incubated overnight to allow attachment. Control cells were processed in parallel. After 48 hours of incubation, cells were washed in pre-warmed XF assay media (or for OCR measurement, XF assay media supplemented with 10mM glucose, 1mM Pyruvate, 2mM L-glutamine and adjusted at 7.4 pH). Cells were then maintained in 175 μL/well of XF assay media at 37°C, in a non-CO_2_ incubator for 1 hour. During the incubation time, we loaded 25 μL of 80mM glucose, 9μM oligomycin, and 1M 2-deoxyglucose (for ECAR measurement) or 10μM oligomycin, 9μM FCCP, 10μM rotenone, 10μM antimycin A (for OCR measurement), in XF assay media into the injection ports in the XFe-96 sensor cartridge. Data sets were analyzed by XF96 software and GraphPad Prism software, using one-way ANOVA and Student's *t*-test calculations. All experiments were performed in quintuplicate, three times independently.

### ATP and glutathione levels

Cell activity was measured using a CellTiter-Glo™ assay kit (Promega, Madison, WI #G7571) and GSH/GSSG-Glo™ Assay kit (Promega, Madison, WI #V6612) following the manufacturer's protocol. For ATP detection, 2 × 10^4^ cells were seeded in a 96-well plate and incubated for 24h. After 24 hours, Celltiter-Glo reagent was added to each well, and the plate was incubated at room temperature for 10 minutes with constant shaking before detected the luminescence intensity. For GSH/GSSH ratio content, 1 × 10^4^ cells were seeded in a 96-well plate and incubated for 24h. After 24 hours, Total and Oxidized Glutathione reagents were added to each well and incubated for 30 minutes. Luciferin was added to each well and the plate was read after 15 minutes. Luminescence intensity was read using a FluoStar Omega plate reader (BMG Labtech) taking the luminescence reading of control as the 100% value. Background measurements were subtracted from all values. The experiments were performed using 4 × 10^4^ cells also and 48 hours of incubation. The results followed the same trend (data not shown). All experiments were performed in sixtuplicate, four times independently.

### Mitochondrial staining

Mitochondrial activity was assessed with MitoTracker Orange (#M7510, Invitrogen), whose accumulation in mitochondria isdependent upon membrane potential. Mitochondrial mass was determined using MitoTracker Deep-Red (#M22426, Invitrogen), localizing to mitochondria regardless of mitochondrial membrane potential. 2 × 10^5^ cells were seeded in a 6 well plate and leave at 37°C for 72 hours. Control cells were processed in parallel. After 72 hours, cells were incubated with prewarmed MitoTracker staining solution (diluted in PBS/CM to a final concentration of 10 nM) for 30-60 minutes at 37°C. All subsequent steps were performed in the dark. Cells were washed in PBS, harvested, and re-suspended in 300 μL of PBS/CM. Cells were then analyzed by flow cytometry. Data analysis was performed using FlowJo software.

### Statistical analysis

Data is represented as the mean ± standard deviation (SD), taken over ≥ 3 independent experiments, with ≥ 3 technical replicates per experiment, unless otherwise stated. Statistical significance was measured using the analysis of variance (ANOVA) test or student *t*-test. *P* ≤ 0.05 was considered significant and all statistical tests were two sided. Mitochondrial staining dats are represented as the mean ± standard deviation (SEM), taken over ≥ 3 independent experiments, with ≥ 3 technical replicates per experiment, unless otherwise stated. Statistical significance was measured using the analysis of variance (ANOVA) test. *P* ≤ 0.05 was considered significant and all statistical tests were two sided.

### Kaplan-Meier (K-M) analysis

To perform K-M analysis on NQO1, NQO2 and GCLC gene transcripts, we used an open-access online survival analysis tool to interrogate publically available microarray data from > 5,000 breast cancer patients. This allowed us to determine their prognostic value. For this purpose, we primarily analyzed data from ER(+) patients that were LN(+) at diagnosis and were of the luminal A sub-type, that were primarily treated with tamoxifen and not other chemotherapy (*N* = 152 patients). Biased and outlier array data were excluded from the analysis. Hazard-ratios were calculated, at the best auto-selected cut-off, and p-values were calculated using the logrank test and plotted in R. K-M curves were also generated online using the K-M-plotter (as high-resolution TIFF files), using univariate analysis: http://kmplot.com/analysis/index.php?p = service&cancer = breast. This allowed us to directly perform *in silico* validation of these biomarker candidates. The most updated version of the database was utilized for all these analyses.

## SUPPLEMENTARY MATERIALS FIGURES AND TABLES


