# Supplementary material for: Mitochondrial “power” drives tamoxifen resistance: NQO1 and GCLC are new therapeutic targets in breast cancer
Source: Oncotarget. 2017 Mar 2;8(12):20309–27. doi: 10.18632/oncotarget.15852 (PMC5386764; doi:10.18632/oncotarget.15852)
Supplement: Supplementary file 1 [file oncotarget-08-20309-s001.pdf]

Mitochondrial “power” drives tamoxifen resistance: NQO1 and GCLC are new therapeutic targets in breast cancer

Supplementary Material

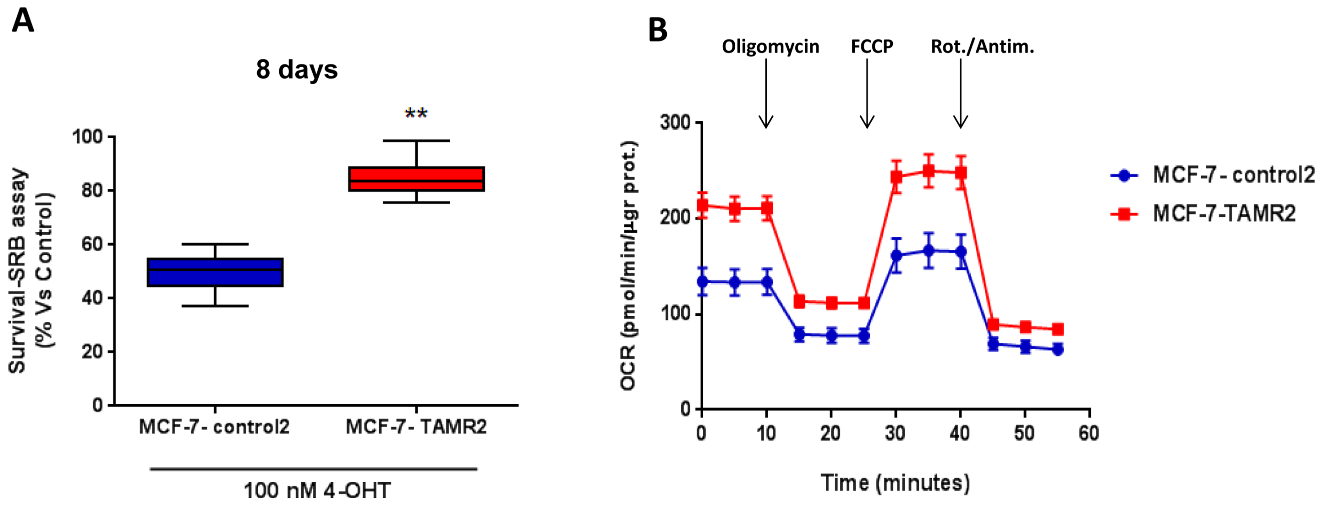

Supplementary Figure 1: Validation of tamoxifen-resistance in a second MCF-7-TAMR cell line. (A) Growth of MCF-7-control2 and MCF-7-TAMR2 cells in the presence of 4-OH-Tamoxifen (4-OHT) 100nM after 8 days from initial treatment. 5 x 10^3 cells were seeded in 96 well plate and treated with 4-OH-Tamoxifen. After 8 days, the SRB assay was performed (see Material and methods). The results for the graphs are expressed as the mean (+/- SD) of six wells repeated three times. * p< 0.01. (B) The Seahorse XF96 analyzer was employed to determine the OCR trend of MCF-7-control2 cells and MCF-7-TAMR2 after 48 hours of treatment with 100nM of 4-OH-Tamoxifen (see Material and methods). The graph shows increased mitochondrial function, as we also demonstrated with MCF-7-control and MCF-7-TAMR (see Figure 2). A representative line graph of 3 independent experiments is shown.

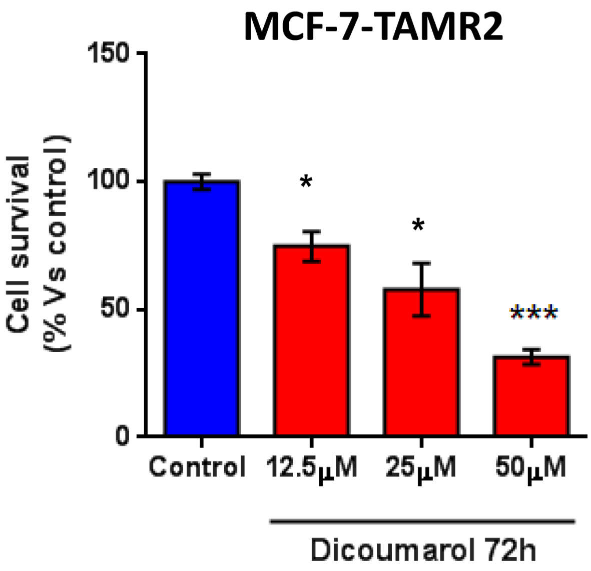

Supplementary Figure 2: TAMR cells are sensitive to dicoumarol, a known NQO1 inhibitor: Effect on survival. Cell survival bar chart of MCF-7-TAMR2 cells treated with increasing concentrations of dicoumarol after 72h of incubation (previous screening showed toxic effect from 12.5 µM to 50 µM). The results are expressed as the mean (+/-SD) of six wells repeated three times. * p< 0.01, *** p< 0.0001.

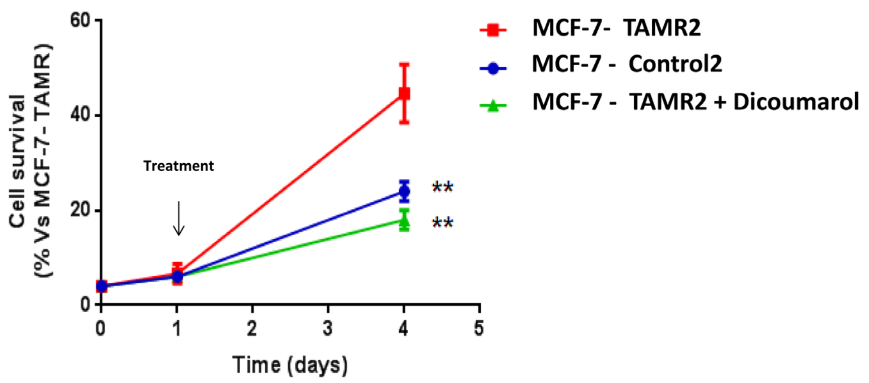

Supplementary Figure 3: TAMR cells are sensitive to dicoumarol, a known NQO1 inhibitor: Effects on cell growth. Growth response of MCF-7-TAMR2 cells incubated in the presence or absence of dicoumarol at 25μM. The growth of tamoxifen-sensitive parental MCF7 cells is also shown for comparison. All three cell lines cells were grown in the presence of 4-OH-Tamoxifen (4-OHT). The arrow indicates the day that fresh growth media containing 4-OHT was added to the cell cultures, in presence or absence of dicoumarol. The results are graphically expresssed as the mean (+/-SD) of six wells repeated three times. ** p< 0.001. Note that treatment with dicoumarol confers tamoxifen-sensitivity and normalizes TAMR cell growth to control levels.

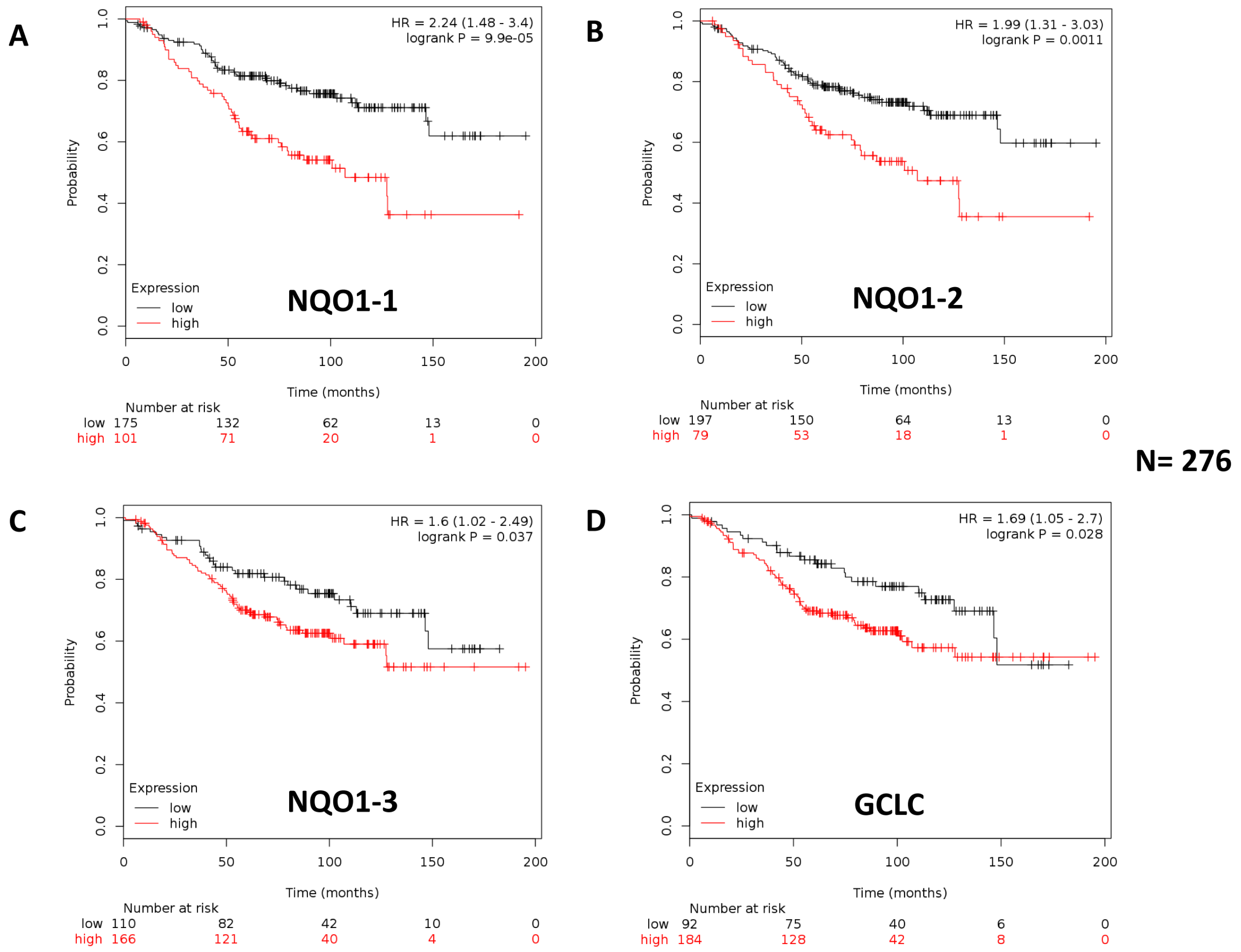

Supplementary Figure 4: Kaplan-Meier (K-M) analysis of the prognostic value of NQO1 and GCLC in ER(+) breast cancer patients receiving endocrine therapy: Luminal B subgroup. Results of recurrence-free survival analysis (RFS) are shown, over a >15-year period of follow-up, for NQO1 (3 independent probes; A-C), and GCLC (D), for ER(+) breast cancer patients (N = 276), with the luminal B sub-type. These patients received endocrine therapy (mostly tamoxifen), but not chemotherapy. Note that higher levels of NQO1 (A-C) and GCLC (D) mRNA species are significantly associated with tumor recurrence. See also Supplemental Table S1.

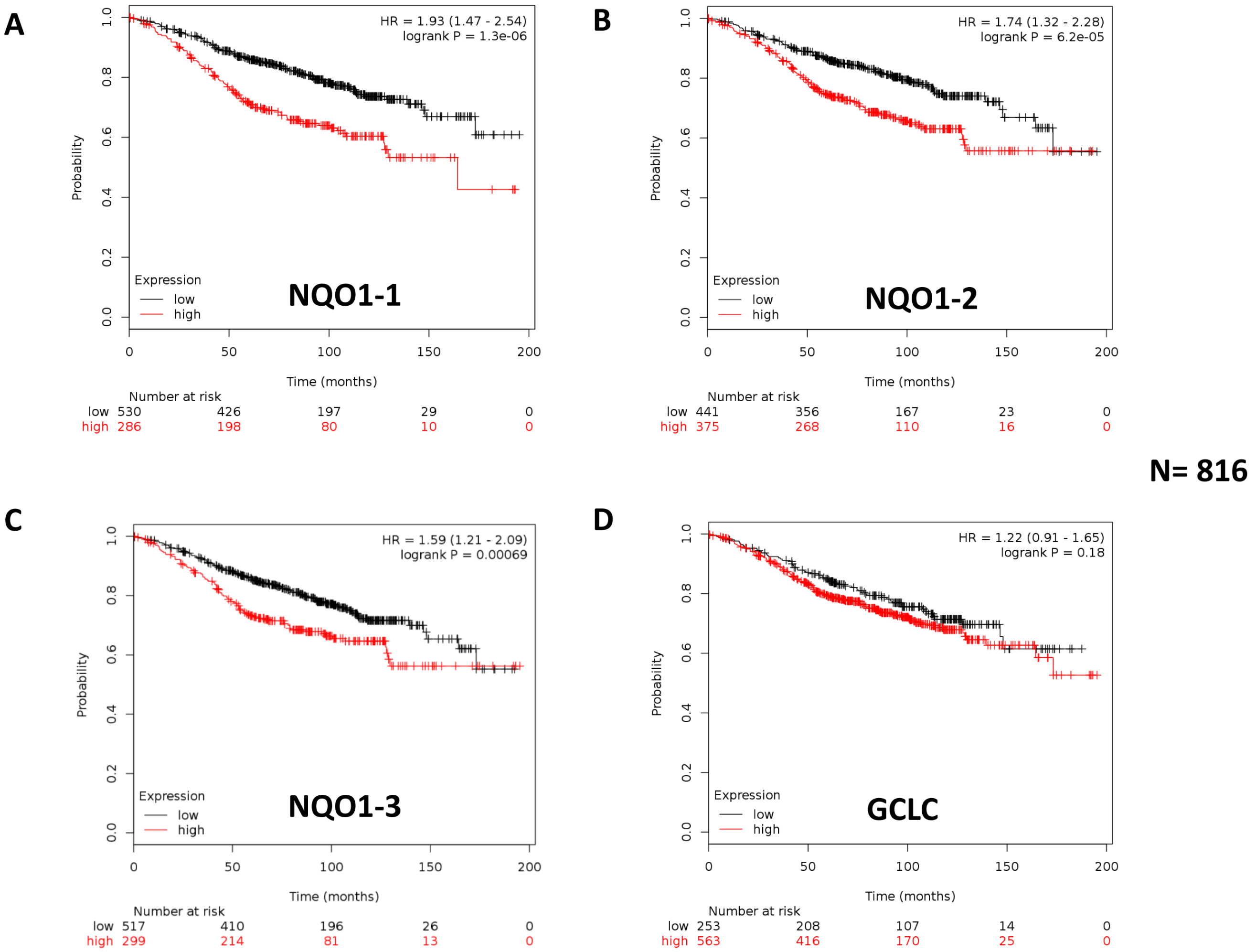

Supplementary Figure 5: Kaplan-Meier (K-M) analysis of the prognostic value of NQO1 and GCLC in all ER(+) breast cancer patients receiving endocrine therapy. Results of recurrence-free survival analysis (RFS) are shown, over a >15-year period of follow-up, for NQO1 (3 independent probes; A-C), and GCLC (D), for all ER(+) breast cancer patients (N = 816). These patients received endocrine therapy (mostly tamoxifen), but not chemotherapy. Note that higher levels of NQO1 mRNA species (A-C) are significantly associated with tumor recurrence. See Supplemental Table S2.

Supplementary Table S1. Prognostic Value of NQO1, NQO2 and GCLC in a High-Risk Subgroup of ER(+) Patients: Luminal B.

Symbol	Probe Number	Hazard Ratio (HR)	P-Value (LogRank)
NQO1-1	201467_s_at	2.24	9.9e-05
NQO1-2	201468_s_at	1.99	0.0011
NQO1-3	210519_s_at	1.60	0.037
NQO2	203814_s_at	1.33	0.25
GCLC	202923_s_at	1.69	0.028

Recurrence-free survival (RFS) in a high-risk population of breast cancer patients: ER(+)/Luminal B/EndocrineTx (mostly Tamoxifen)/**N=276 patients.**

Supplementary Table S2. Prognostic Value of NQO1, NQO2 and GCLC in all ER(+) Breast Cancer Patients.

Symbol	Probe Number	Hazard Ratio (HR)	P-Value (LogRank)
NQO1-1	201467_s_at	1.93	1.3e-06
NQO1-2	201468_s_at	1.74	6.2e-05
NQO1-3	210519_s_at	1.59	0.00069
NQO2	203814_s_at	1.33	0.086
GCLC	202923_s_at	1.22	0.18

Recurrence-free survival (RFS) in a population of breast cancer patients: ER(+)/EndocrineTx (mostly Tamoxifen)/**N=816 patients.**
